# Supplementary material for: Study examining the significant role of intellectual property protection in driving radical technological innovation among national research project teams, employing PLS-SEM and ANN modeling
Source: PLoS One. 2024 Aug 7;19(8):e0307026. doi: 10.1371/journal.pone.0307026 (PMC11305571; doi:10.1371/journal.pone.0307026)
Supplement: Attachment — Submitted filename: Response to Reviewers.pdf [file pone.0307026.s003.pdf]

The Response to Reviewers for Major Revision

Study examining the significant role of intellectual property protection in driving radical technological innovation among national research project teams, employing PLS-SEM and ANN modeling

To Chief Editor and Reviewer

We are grateful for the opportunity to submit a revised draft of the manuscript "Study examining the significant role of intellectual property protection in driving radical technological innovation among national research project teams, employing PLS-SEM and ANN modeling" for publication in the PLOS ONE. We appreciate the time and effort that editors and the reviewers dedicated to providing feedback on our manuscript and are grateful for the insightful comments and valuable improvements to our paper. We have incorporated most of the suggestions made by the reviewers. Please see below, for a point-by-point response to the re- viewer' comments and concerns. All section numbers refer to the revised manuscript file with tracked changes.

Reviewer #1

The Reviewer's Comment #1.1

The manuscript is well structured, it is suggested that kindly run grammarly for checking any typos and errors. Also, try to enhance the language of paper by using academically mature words so that the manuscript shall look sound enough to the readers.

Response:

Thank you very much for your nice reminder. We value your detailed feedback on enhancing the linguistic quality of our manuscript. Following your advice, we have carefully reviewed and revised the language and vocabulary of the paper. We collaborated with two doctoral candidates and two master's students specializing in English to meticulously scrutinize and refine each sentence. We believe these efforts have significantly improved the manuscript, making it more academically robust and appealing to our readers. All specific modifications have been annotated in the revised manuscript file with tracked changes.

Reviewer #2

The Reviewer's Comment #2.1

I am delighted to review the article entitled "Study examining the significant role of intellectual property protection in driving radical technological innovation among national research project teams, employing PLS-SEM and ANN modeling." Authors have chosen a very interesting topic, but there are some areas which need to be corrected. There are numerous repeated abbreviations and annotations in the text that require modification. When the words first appear, you can abbreviate the remarks, and you can use abbreviations directly afterwards.

Response:

Thank you very much for pointing this out. We have thoroughly reviewed the abbreviations and notes in this article. As suggested, we introduced abbreviations at their first occurrence and subsequently used these abbreviations throughout the text. All specific modifications have been annotated in the revised manuscript file with tracked changes.

The Reviewer's Comment #2.2

The authors need to provide a more detailed literature review of the study, such as a literature review on national research project teams in driving innovation-driven growth in IPP and technological progress in previous studies. Other literature reviews should also include IL, IM, RD&II, and GP, but the assumptions section only includes a few. Relevant literature needs to be presented in the table form.

Response:

Thank you very much for your nice reminder. We have expanded the literature review section to include a detailed analysis of IPP, IL, IM, RD&II, GP, and RTI, along with their direct connections, within the theoretical frameworks and hypotheses section. We have extensively reviewed and augmented the relevant literature to strengthen the theoretical foundation of this study. We value your suggestion to summarize the key points of the literature in a table format, which we acknowledge can help readers visualize the perspectives of various scholars. However, in our discussion section, we opted to enhance organization and readability by providing a detailed narrative review of the newly added literature, aiming for clarity and intuitiveness. Consequently, we did not include a tabular presentation of reference narratives in the manuscript. The revision is in Section 2 [Ln145-146,148-149,152-156,164-172,185-186,187-190,216-223,224-226,237-239,241-242,250-260,265-269,281-284,300-301,312-313,319-323,338-343,348-352].

"⋯ Research indicates that IPP offer both positive external incentives and negative external challenges for RTI. ⋯"

"⋯ Enhanced IPP allows national research program teams to financially benefit from RTI, thereby enabling further investment in research and development (R&D) activities (Qayyum et al., 2022). ⋯"

"⋯ IPP enhance the exclusivity of knowledge through legal measures, which amplifies the monopoly power of innovators. This elevation in monopoly power significantly increases the discounted value of expected profit streams from innovations, thereby directly stimulating innovation incentives (Bican et al., 2017; Hudson and Minea, 2013; Lee et al., 2018; Song et al., 2024). IPP enhances green innovation in corporate research teams by mitigating R&D spillover losses and easing financing constraints (Gao & Zhao, 2023). ⋯"

"⋯ National research project teams transcend organizational boundaries to harness external technological resources and knowledge, thereby accelerating internal innovation, enhancing inter-organizational collaboration, and fostering RTI (Chesbrough, 2003c; Teece, 2018; Dahlander & Gann, 2010; Vanhaverbeke & Cloodt, 2014). Through technological development, organizations integrate externally acquired technologies with internal resources and capabilities, generating new knowledge that benefits partners and catalyzes future collaborations (Dahlander & Gann, 2010; Obradovic et al., 2021). Conversely, the IPP system, by establishing a legally protected monopoly, restricts knowledge spillovers and complicates the accumulation of knowledge, thus impeding cumulative innovation (Lorenczik & Newiak, 2012; Schwarz, 2019). Moreover, IPPs strengthen the monopoly power of IPP owners and diminish the incentives for open competition and R&D investment, ultimately discouraging innovation (Chirico et al., 2020; Hwang et al., 2016). ⋯"

"⋯ Grimaldi et al. (2021) assert that no IPP strategy significantly impedes the success of RTI. ⋯"

"⋯ Hagedoorn and Zobel (2015) observe that many firms in developed countries across Europe and North America regard formal contractual agreements as crucial legal mechanisms for safeguarding intellectual property rights in collaborations with Open Innovation partners. ⋯"

"⋯ This leadership style represents a new paradigm emerging from the integration of leadership and innovation, characterized by integrative leaders who possess distinct qualities that allow them to identify and leverage environmental opportunities (Khanjanian & Olfati, 2019). It is posited that IL is intrinsically linked to opportunity identification (Renko, 2015). A fundamental aspect of entrepreneurial leadership involves embracing the concept of technological innovation, which connects breakthrough opportunities with potential markets (Lam et al., 2021; Ta'Amnha et al., 2023). The success of RTI hinges on the mindset and culture of leaders and actors, who must maintain a dynamic approach to opportunities and possess the requisite skills for their interpretation (Freixanet et al., 2021; Priyono & Hidayat, 2022; Osobajo et al., 2023). ⋯"

"⋯ IL significantly contributes to the development of RTI by facilitating these processes. Numerous studies have demonstrated that IL is a significant motivator in RTI (Swiercz & Lydon, 2002; Freeman & Siegfried Jr, 2015; Karol, 2015; Bagheri, 2017; Esmer & Dayi, 2017). ⋯"

"⋯ Integrative leaders significantly influence subordinates' work-related behaviors and attitudes within the workplace (Shalley, 2008), and they critically impact self-efficacy and creativity (Tierney, 2002; Lee,

2019). …"

"… Mutual respect and service, fundamental to an IL approach, enhance innovation self-efficacy, which in turn boosts innovation effectiveness (Suhartanti, 2022). …"

"… IPP can indirectly influence the IM within teams, impacting trust both positively and negatively. The economic theory of cost transactions posits that trust acts as a substitute for contracts (Hosmer, 1995). An increased reliance on contracts might diminish trust, as contracts may signal a lack of trust and the anticipation of opportunistic behavior, subsequently fostering an unfavorable IM (Gallivan & Depledge, 2003; Kadefors, 2004; Mayer & Argyres, 2004). Kadefors (2004) discovered that detailed contract specifications and stringent monitoring negatively affect both trust and team climate. Conversely, less detailed contracts can function as a trust mechanism, fostering trust by clarifying expectations, roles, and responsibilities (Mayer & Argyres, 2004). Woolthuis, Hillebrand, and Nooteboom (2005) observed that IPR protection and IM mutually enhance each other in project teams. IPR protection mechanisms safeguard proprietary knowledge (Olander et al., 2010), thus reducing the risk and fear of exploitation. Such protection can bolster stability and predictability (Olander et al., 2010), and positively affect the team's IM(Argyres, Bercovitz, & Mayer, 2007; Blomqvist, Hurmelinna, & Seppänen, 2005). …"

"… RTI are enabled by employees who creatively use their knowledge and skills to integrate resources, benefiting the national research project team (Lusch & Nambisan, 2015). This integration significantly impacts the outcomes of technological innovations and the realization of radical advances (Ordanini & Parasuraman, 2011). Furthermore, Lusch and Nambisan (2015, p. 157) contend that effective communication among team members facilitates the sharing and integration of resources and knowledge, which in turn promotes RTI. …"

"… Knowledge production within national research project teams hinges on the integration of insights from various fields and disciplines, with interorganizational teamwork serving as a vital conduit for achieving efficient and effective organizations (Newell, Robertson, Scarbrough, & Swan, 2009; Shalley & Gilson, 2004). Additionally, safeguarding the workflows of interorganizational teams is crucial for fostering knowledge sharing and innovation (Rosendahl, Olaisen, & Revang, 2014). …"

"… Venture capitalists fund the development of promising inventions, thus advancing technological innovation and potentially impacting firms' patenting activities (Arqué-Castells, P., 2012). …"

"… Sun et al. (2024) demonstrate that increased investment in R&D indirectly fosters regional green technology innovation, influenced by digital financial inclusion. …"

"… R&D encounters negative externalities as national research program teams often work to prevent the imitation of their intellectual property. Enhanced governmental IPP mitigates these externalities by reducing the risk of IPP infringement and boosting the expected returns on R&D investments, thereby motivating countries and firms to augment their R&D efforts (Czarnitzki et al., 2015; Kanwar, 2007; Zhao, 2006). However, this also compels firms to enhance their competitive edge and increase their innovation-driven

competitiveness (Aggarwal & Hsu, 2014). ⋯"

"⋯ The creativity and creative potential of researchers are crucial prerequisites for innovation. This potential is influenced not only by individual creativity but also by environmental factors (Salas et al., 2004). Therefore, creating an environment that stimulates the innovative potential of research teams is a vital component of their innovation management. Research indicates that executives proficient in digital literacy and regions with robust IPP significantly enhance the positive effects of digital transformation on the quality of technological innovation (Huang & Lau, 2024). ⋯"

"⋯ Research indicates that GP significantly impacts team success in achieving work objectives (Gibson, 1999). Furthermore, GP exerts a more direct and potent influence on team empowerment and performance than individual efficacy (Kirkman and Rosen, 1999). National research teams, fueled by this GP, conduct research and innovation through effective integration of internal factors, thereby enhancing their collective research and innovation capabilities. ⋯"

The Reviewer's Comment #2.3

In the discussion, considering the different methods of PLS-SEM and ANN, are the key predictive factors obtained consistent or inconsistent? The discussion did not include the comparison results of the two methods.

Response:

Thanks for your nice reminder. In this study, the key predictors identified by both the PLS-SEM and the ANN models are consistent. We have included a comprehensive description and comparative analysis of the results in the Structural Model and ANN Analysis sections. Additionally, we have incorporated a comparative evaluation of the PLS-SEM and ANN models in the Discussion section. The revision is in Section 4 and Section 5 [Ln606-608,660,700-703,704-706,718-720].

"⋯ Among the hypothesized relationships, the most significant factor directly affecting RTI is the R&DII, which exhibits a 0.534 percent increase for every 1 percent increase in R&DII ⋯"

"⋯ The results from the ANN model align with those derived from the PLS-SEM model. ⋯"

"⋯ ~~The results of the hybrid SEM-ANN analysis indicate that R&DII is a vital predictor of RTI in national research project teams (H4b),~~ The results from the PLS-SEM analysis align with those from the ANN analysis, demonstrating that the R&DII is the most significant predictor of RTI in national research project (H4b). ⋯"

"⋯ The findings confirm that the linear relationships identified by the PLS-SEM are corroborated by the nonlinear relationships discerned through the ANN model. ⋯"

"⋯ In this study, the analysis of both the PLS-SEM and the ANN consistently indicates that the key predictors

of RTI in national research project teams are ranked in descending order of importance as follows: R&DII, IL, IM, IPP, and GP. …"

The Reviewer's Comment #2.4

Solving CMB with marker variables allows for a comparison of path efficiency (β) and p value. You could include a description in this section to see if the changes in the path coefficient and p-value are within 10%.

Response:

Thank you very much for your nice reminder. In the Common Method Bias section, we detailed the path coefficients, p-values, and R^2 values of the conceptual model without labeling variables, compared to those of the model with labeling variables. The variations between the two were found to be within 10%. To enhance reader comprehension, we included a comparison table that visually represents these differences.The revision is in Section 3 [Ln467-473,821-822].

"… ~~The analysis by Lindell and Whitney (2001) revealed minor and non-significant correlations (ρ=0.01-0.04), indicating the absence of a common methodological bias in the study's data. This implies that the data in this study might have been free from common methodological biases.~~ As indicated in Table 4, the path coefficients, p-values, and R^2 values of the conceptual model without marker variables show no significant differences compared to the model with marker variables, as variations are within 10% (Chin et al., 2013). Consequently, it can be concluded that CMB did not affect the study's outcomes (Lindell & Whitney, 2001). [Table 4 Measured marker variable test.] …"

Table 4 Measured marker variable test.

	Baseline model		Marker variable model	
**Structural Path**	**Coefficient**	**P-values**	**Coefficient**	**P-values**
IPP -> RTI	0.153	0.013	0.160	0.003
IPP -> IL	0.021	0.000	0.020	0.000
IL -> RTI	0.420	0.000	0.407	0.000
IPP -> IL -> RTI	0.009	0.000	0.008	0.001
IPP -> IM	0.029	0.013	0.021	0.024
IM -> RTI	-0.199	0.008	-0.219	0.001
IPP -> IM -> RTI	-0.006	0.046	-0.004	0.048
IPP -> RDII	0.018	0.001	0.019	0.001
RDII -> RTI	0.534	0.000	0.481	0.000
IPP -> RDII -> RTI	0.010	0.003	0.009	0.002
GP x IL -> RTI	0.121	0.039	0.110	0.022

GP x RDII -> RTI	-0.167	0.005	-0.157	0.003
**R Square**				
IPP	1		1	
RTI	0.718		0.727	
IL	0.997		0.997	
IM	0.982		0.983	
R&DII	0.993		0.993	

Source: Authors' calculation.

Reviewer #3

The Reviewer's Comment #3.1

An interesting topic but few aspects need to be revisit. The specific research question and objectives need to be improved but make it simple.

Response:

Thank you very much for your nice reminder. We have streamlined Hypotheses H1, H5a, and H5b to enhance their intuitiveness. The revision is in Section 2 [Ln194-196,362-369].

"··· ~~**H1**: IPP mitigates spillover effects, significantly enhancing the innovation capabilities of national research project teams.~~**H1**: IPP significantly enhances RTI within national research project teams. ···"

"··· ~~**H5a:** The GP of the national research project team is able to moderate the mediating role played by IL in the impact of IPP on RTI.~~**H5a:** GP acts as a moderating factor, while IL serves as a mediating factor in the impact of IPP on RTI within national research program teams. ···"

"··· ~~**H5b:** The GP of the national research project team is able to moderate the mediating role played by R&DII in the impact of IPP on RTI.~~**H5b:** GP acts as a moderating factor, while R&DII serves as a mediating factor in the impact of IPP on RTI within national research program teams. ···"

The Reviewer's Comment #3.2

Need elaboration on how the underpinning theories help to explain the phenomena of the study/gap.

Response:

Thanks for your nice reminder. In the Theoretical Frameworks and Hypotheses section, we expand upon the innovation-driven theory and the Ability-Motivation-Opportunity theory. The innovation-driven theory is discussed in detail, highlighting its macro perspective on the effects of implementing IPP systems on RTI within national research project teams. Additionally, we explore RTI's influences from an intra-team viewpoint, employing the micro perspective offered by AMO theory, which is elaborated to analyze team dynamics. The

revision is in Section 2 [91-143,199-207].

"··· In order to delve deeper into the influence mechanisms between IPP and RTI in national scientific research projects, this study utilizes the innovation-driven theory outlined in Michael Porter's book "National Competitive Advantage." The theory suggests that a country's economic development must progress through four stages: factor-driven, investment-driven, innovation-driven, and wealth-driven. By leveraging technology, knowledge, system, and management as the primary drivers of innovation, this approach optimizes resource allocation by utilizing new knowledge and technology, enhances labor quality, and improves the scientific research environment, thereby fostering continuous innovation.(Adisu et al. , 2023). Drawing upon Appelbaum's AMO theory (2000), the fundamental principle is that the team's overall well-being is optimized when its members' competence, motivation, and opportunities are addressed. To achieve this, strategies are implemented to equip team members with the necessary information and skills, encompassing recruitment, selection, development, and training programs. Incentives encompass both monetary and non-monetary rewards, based on performance evaluations, and serve to motivate individuals towards achieving their goals. Opportunities are generated by policies and regulations, which enhance employee participation in a variety of activities through improving interaction, promoting information sharing, and ensuring personal autonomy. This research, drawing on the innovation-driven theory and the AMO theory, argues that the IPP system implemented by the government plays a crucial role in promoting technological innovation. Furthermore, a comprehensive analysis of the IPP's influence is required by incorporating relevant factors, such as IL, IM, GP, and R&DII, to assess the full extent of its impact on the Research and Technology Innovation (RTI) within national scientific research projects.To better elucidate the influence between IPP and RTI in national research programs, this study utilizes Michael Eugene Porter's innovation-driven theory and Appelbaum's Ability-Motivation-Opportunity (AMO) theory to examine how IPP can foster breakthroughs in technological innovations within research teams (Porter, 2011; Appelbaum, 2000). Porter's theory delineates four pivotal stages of national economic development: factor-driven, investment-driven, innovation-driven, and wealth-driven. It emphasizes that the innovation-driven stage enhances innovation levels by leveraging technology, knowledge, systems, and management as primary drivers, thus optimizing resource allocation, enhancing labor quality, and improving the research environment (Adisu et al., 2023). IPP enhances firm productivity by stimulating technological innovation within research teams, improving investment efficiency, and bolstering competitive advantage (Li, 2024). Barrios et al. (2024) highlight that the interplay between open innovation and the use of non-disclosure agreements for protecting intellectual property rights can influence the innovation performance of manufacturing and service firms. Conversely, AMO theory suggests that satisfying team members' abilities provides them opportunities to optimally utilize their skills. By meeting the ability, motivation, and opportunity needs of team members, maximal team benefits are realized. These frameworks were chosen for their robust analytical structure, enabling a deep understanding of research innovation complexities,

particularly through the lens of institutional factors and team dynamics. Innovation-driven theory underscores the essential role of knowledge and technology in economic growth, highlighting the critical importance of intellectual property regimes in sustaining innovation (Kalanje et al., 2006). Meanwhile, AMO theory offers a micro-level perspective that provides a practical framework for analyzing and influencing team dynamics, detailing how competencies, incentives, and opportunities interact at the team level to impact organizational performance (Bos-Nehles et al., 2023). ⋯"

"⋯ This study aims to address a critical gap in existing research, which broadly acknowledges that IPP stimulates innovation but often lacks comprehensive analysis of its effects on the innovation processes within research teams. Utilizing the analytical framework of the Ability-Motivation-Opportunity (AMO) theory, this paper investigates how IPP fosters innovation by enhancing researchers' motivation (e.g., through patent incentives and increased R&D funding), empowering them via training and technical support, and creating opportunities for high-quality innovation outcomes (e.g., by promoting cross-disciplinary collaborations and enhancing integrative leadership). For instance, IPP increases researchers' incentives to engage in high-risk projects by ensuring commercial returns on research results; it strengthens research teams' access to critical technological resources through institutional mechanisms (Williams et al., 2013); and it facilitates cross-disciplinary collaboration that enhances team communication and cooperation (Cho et al., 2015). This integration of theory not only deepens our understanding of IPP's role in research teams but also offers actionable insights for enhancing research management and policy-making, thus providing substantial theoretical and practical contributions to the literature. By examining the multifaceted impacts of IPP, this study enriches the existing literature and lays a scientific foundation for developing more effective policies to promote innovation. ⋯"

"⋯ The current IPP regime is theoretically justified largely on moral grounds derived from utilitarianism, and theories of labor and personhood, which emphasize private ownership and the maximization of economic profits (Ballardini et al., 2021; Pihlajarinne & Ballardini, 2019). This foundation aligns consistently with systemic cooperation in RTI, particularly regarding innovation goals that encompass economic, cultural, social, and environmental sustainability dimensions. It also underscores the necessity of reciprocity and collective efforts to achieve co-specialization in shared technologies (Witoszek & Sorensen, 2018). For instance, RTI relies not only on sharing IPP innovations and technologies but also on diversifying and integrating knowledge among leaders and stakeholders to manage conflicts of interest and maintain productive cooperation that leverages the potential of IP (Andersen & Konzelmann, 2008). ⋯"

The Reviewer's Comment #3.3

Further explanations are required why ANN analysis are needed? How it help to achieve the research gaps?

Response:

Thank you very much for your nice reminder. In the Research Methods, Results, and Discussion sections, we have incorporated a detailed explanation of the necessity for ANN analysis. This inclusion elucidates how ANN enhances data analysis and result prediction, thereby increasing the precision and accuracy of the study. The revision is in Section 3, Section 4, Section 5 [Ln498-504,505-507,622-630,706-711].

"··· Furthermore, ANN are resilient to outliers in small samples and accommodate non-compensatory models, where a decrease in one variable does not necessitate a corresponding increase in another (Leong et al., 2020; Chahal et al., 2023). This method enables researchers to initially apply the PLS-SEM estimation to establish linear relationships between predictors and specific target factors (Ahmed and Hashim, 2022; Bhutto et al., 2023). Subsequently, based on these results, the ANN technique is employed to explore non-linear relationships (Ibrahim et al., 2023). The hybrid SEM-ANN approach proves effective for human behavioral studies involving complex processes and data, as demonstrated by the current study (Hidayat-ur-Rehman et al., 2022; Almarzouqi et al., 2022; Yang et al., 2022). ···"

"··· The results of the ANN model were analyzed and compared with those from the PLS-SEM to assess the consistency of the predictors' relative importance derived from both methods, ensuring high accuracy. ···"

"··· The PLS-SEM model does not address nonlinear relationships (Alnoor et al., 2024). Consequently, an ANN analysis was employed both to validate the PLS-SEM results and to explore nonlinear associations. The integration of PLS-SEM with ANN aims to demonstrate that relationships are not only linear but also compensatory (Sabri et al., 2023). Additionally, ANN deliver highly accurate results by detecting linear and nonlinear correlations among variables (Alnoor et al., 2024). ANN acquire knowledge through iterative training, analogous to the human brain, and encode this knowledge in synaptic weights (Leong et al., 2020). An activation function regulates these weights, minimizing the discrepancy between the actual and desired outputs through iterative adjustments. Researchers have applied ANN techniques across various fields, including accounting (Moussaid et al., 2023; Parot et al., 2019), management (Alnoor et al., 2024), and tourism (Teixeira and Fernandes, 2012). ···"

"··· These results provide more substantive insights than previous studies, which solely focused on linear relationships between structures (Yilmaz & Ari, 2017; Shen et al., 2016; Yuan et al., 2019; Zhang et al., 2019). Consequently, this research underscores the theoretical implications of employing the SEM-ANN predictive analytics framework, a novel methodological paradigm within transportation engineering literature. Moreover, this study reveals a nonlinear relationship between the predictor and target structures, reinforcing the validity of the linear analyses conducted using PLS-SEM. ···"

The Reviewer's Comment #3.4

Lack of measurementdetails and references.

Response:

Thank you very much for pointing this out. In the Research Methods and Results sections, we offer a detailed description of the measurement techniques and enhance the theoretical support by incorporating additional references. The revision is in Section 3, and Section 4 [Ln389-390,559-568,569-572,634-635,639-642,648-651].

"··· This study employed three trained researchers to collect data using a random selection method. ···"

"··· In structural equation modeling, the model's predictive power is evaluated based on two criteria: predictive accuracy and predictive relevance. Hair et al. (2014) and Khan, Hashim, and Bhutto (2021) clarified that predictive accuracy assesses the explained variance of each endogenous construct using the coefficient of determination (R^2). R^2 and Q^2, as indicators of predictive power, are employed respectively according to Cohen (1988); a value of R^2exceeding 0.26 is considered valuable. The R^2 values for the variables IPP, RTI, IL, IM, and RD&II in Table 6 are 1, 0.718, 0.997, 0.982, and 0.993, respectively, indicating the variables' strong predictive abilities (Shen, 2016; Ibrahim, 2022; Waris et al., 2024). Additionally, Q^2's magnitude signifies the importance of the endogenous component. This study was evaluated using the Q^2 value, calculated through the blindfolding procedure in SmartPLS 4.0 software, with a value greater than 0 indicating its relevance in prediction (Henseler 2016). ···"

"··· This indicates that the model's predictive relevance is adequate (Ibrahim, 2023). Additionally, the SRMR values from the PLS-SEM were utilized to evaluate the model's fit, and the SRMR coefficient for the model in this research was 0.053, which fell short of the 0.1 threshold. Consequently, the model exhibits a satisfactory fit (Tian 2023). ···"

"··· The number of neurons in the hidden layer is autonomously generated by the SPSS neural network module (Kheirollahpour et al., 2020; Leong et al., 2020; Xu et al., 2019). ···"

"··· The data were divided into two segments: 90% for training the model and 10% for testing it (Alkawsi et al., 2021; Leong et al., 2015). The predictive accuracy of the ANN model was assessed using the root mean square error (RMSE) values from both the training and test datasets (Leong et al., 2015; Hyndman & Koehler, 2006). Hyndman and Koehler (2006) note that the RMSE value is always positive, with a value of zero indicating perfect accuracy. ···"

"··· As suggested by Leong et al. (2020), ANN models have excellent predictive accuracy and matching. The RMSE values of the model developed in this study are lower than those reported by predictive models developed in previous studies such as Ibrahim et al. (2023) and Alkawsi et al. (2021) et al. ···"

The Reviewer's Comment #3.5

Lack of critical discussion in the discussion part.

Response:

Thank you very much for your nice reminder. We have included discussions of aspects previously overlooked by scholars, as well as an analysis of the limitations present in this paper. The revision is in Section 5 [Ln674-677,688-690,727-731].

"⋯ Panpan Zheng et al. (2023) focus their analysis solely on the impact of external institutional factors on technological innovation, considering the internal dynamics of firms and research teams. In contrast, this study broadens the scope by introducing a new model that comprehensively incorporates internal team factors, potentially influencing RTI. ⋯"

"⋯ This study diminishes the direct role of IL in RTI by treating it solely as a mediating variable. However, it expands the understanding of the factors influencing RTI by situating it within a complex framework. ⋯"

"⋯ The factors influencing RTI in national research project teams extend beyond the immediate project parameters to include economic, trade, and market dimensions. Currently, these teams are predominantly based in universities and research institutes, with only a minor presence in enterprises. This represents a gap that future research could address by enhancing the focus on corporate involvement. ⋯"

Reviewer #4

The Reviewer's Comment #4.1

Although the original data has been provided, it was written in Mandarin language. Perhaps, it is best to have an English version too, considering English as an international language and widely spoken among scholars/researchers.

Response:

Thank you very much for your nice reminder. We acknowledge the importance of accessibility and have translated the original data into English, which is now included as an attachment.
